# A Simple and Specific Stability- Indicating RP-HPLC Method for Routine Assay of Adefovir Dipivoxil in Bulk and Tablet Dosage Form

**Published:** 2017

**Authors:** Bahar Darsazan, Alireza Shafaati, Seyed Alireza Mortazavi, Afshin Zarghi

**Affiliations:** a *Department of Pharmaceutics, School of Pharmacy, Shahid Beheshti University of Medical Sciences, Tehran, Iran.*; b *Department of Pharmaceutical Chemistry, School of Pharmacy, Shahid Beheshti University of Medical Sciences, Tehran, Iran.*; c *Students Research Committee, School of Pharmacy, Shahid Beheshti University of Medical Sciences, Tehran, Iran.*

**Keywords:** Adefovir Dipivoxil, Assay, RP-HPLC, Stability-Indicating, Pharmaceutical dosage form

## Abstract

A simple and reliable stability-indicating RP-HPLC method was developed and validated for analysis of adefovir dipivoxil (ADV).The chromatographic separation was performed on a C_18_ column using a mixture of acetonitrile-citrate buffer (10 mM at pH 5.2) 36:64 (%v/v) as mobile phase, at a flow rate of 1.5 mL/min. Detection was carried out at 260 nm and a sharp peak was obtained for ADV at a retention time of 5.8 ± 0.01 min. No interferences were observed from its stress degradation products. The method was validated according to the international guidelines. Linear regression analysis of data for the calibration plot showed a linear relationship between peak area and concentration over the range of 0.5–16 μg/mL; the regression coefficient was 0.9999and the linear regression equation was y = 24844x–2941.3. The detection (LOD) and quantification (LOQ) limits were 0.12 and 0.35 μg/mL, respectively. The results proved the method was fast (analysis time less than 7 min), precise, reproducible, and accurate for analysis of ADV over a wide range of concentration. The proposed specific method was used for routine quantification of ADV in pharmaceutical bulk and a tablet dosage form.

## Introduction

**Figure 1:**
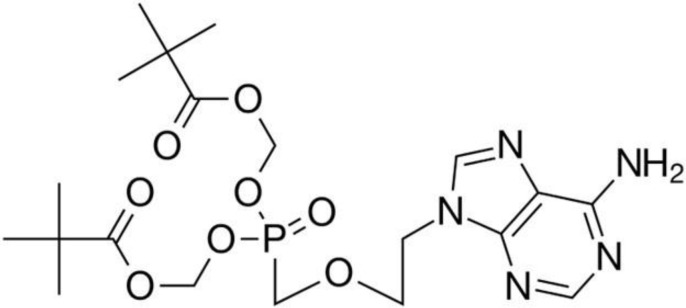
Structure of adefovir dipivoxil

Adefovir dipivoxil (ADV) (Figure 1) or (bis(POM)-PMEA), is known chemically as 9-[2-({bis[(pivalaloyloxy)methoxy]phosphinyl]}methoxy)ethyl]adenine (1, 2). ADV is a diester prodrug of the active moiety adefovir, which is an acyclic nucleotide analogue of adenosine monophosphate (3). ADV is a reverse transcriptase inhibitor used for the treatment of chronic hepatitis B. Following absorption, adefovir dipivoxil is converted into adefovir and then phosphorylated to adefovir diphosphate in hepatocytes, which is a competitive inhibitor of hepatitis B virus (HBV) polymerase (4). Thus, ADV as an antiviral drug is highly efficient in the treatment of human hepatitis B virus (HBV) infections.

Several methods have been reported for the analysis of ADV in bulk and pharmaceutical formulations, including uv-visiblespectrophotometric method for the estimation of the drug and its stress degradation products in bulk and dosage forms (5). Although the method is simple and claimed to be stability-indicating, one could not detect ADV and its stress degradation products in a single analysis run. Also, a high-performance liquid chromatographic (HPLC) method reported for assay of the drug in the pharmaceutical samples (6) without considering the degradation of ADV. Other HPLC methods were also reported for the determination of ADV and its degradation products using a CN-column (7), by applying ion-pair HPLC (8) and by using a C18 column (9). However, these HPLC methods were developed using mobile phases with high content of the organic modifiers to allow concomitant detection of ADV, adefovir monopivoxil and adefovir base. For example, a recently Published work proposed an RP-HPLC method on a C18 column using methanol-buffer at 80:20 (v/v) as mobile phase.

The aim of this study was to develop a simple, rapid and economicalstability-indicating HPLC method with UV detection, specifically designed to assay the drug for a routine quality control method in the industry. 

## Experimental

*Materials*

ADV standard (99.7%) was obtained as a gift sample from Zhejiang Charioteer Pharmaceutical Co. Ltd., (Dazhan, China) and the commercial tablet formulation (Biofovir-10 mg tablet) was obtained from Bakhtar Bioshimi Pharmaceutical Co, (Kermanshah, Iran).HPLC-grade acetonitrile, citric acid and sodium hydroxide were purchased from Merck (Darmstadt, Germany). HPLC grade water used throughout the study was prepared by reverse osmosis and passed through a 0.45 μM Millipore filter (Millipore Company, USA) before use. Other chemicals and reagents were of Merck.

*Chromatographic conditions*

Chromatography was performed, under ambient conditions, with Kanuer-smart line (Germany) HPLC equipment comprising EA4300F pump and E4310 2500 UV-Visible detector. Samples (40 μL) were injected by means of a Rheodyne injector fitted with a 20-μL loop. The instrumentation was controlled by use of EZchrom Elite software. Compounds were separated on a Nucleodur column (manufactured by MN Company) with C_18_ packing and 15 cm × 4.6 mm i.d., 5-μM particle, (Duren, Germany). The mobile phase was acetonitrile-citrate buffer 10 mM (PH = 5.2) 36:64 v/v, respectively, at a flow rate of 1.5 mL/min. The elute was monitored at 260 nm.

The mobile phase was freshly prepared each day and filtered through a 0.45-μM membrane filter.

*Preparation of standard solution*

A stock solution of ADV (100 μg/mL) was prepared by dissolving 50 mg drug in 100 mL of methanol, then transferring 20 mL of this solution to a 100 mL volumetric flask and diluting to the volume by adding the mobile phase. The prepared stock solution was stored at 4 °C in a glass vial. From this stock solution, standard solutions were freshly prepared by adding appropriate amount of the mobile phase prior to analysis.

*Assay of ADV in tablet*

For analysis of commercial formulation, twenty tablets were weighed and crushed into fine powder. An accurately weighed quantity of the powder equivalent to 10 mg of ADV was transferred into a 100 mL volumetric flask containing 10 mL of methanol and the mixture was stirred for 10 min, to ensure the complete dissolution of the drug.The mixture was then made up to 100 mL with citrate buffer of pH 5.2. Five mL of this solution was filtered to a 100 mL volumetric flask and made up to the mark with the buffer. The resulting solution was filtered through a 0.45 μM membrane filter prior to injection into the column. The procedure was repeated 5 times and the mean values of peak areas were calculated and the drug content in the tablets was quantified against a standard solution of ADV, prepared in the same way as described for the commercial tablet.

*Degradation studies*

The forced degradation of the drug was carried out (10) with 0.1 M HCl, 0.001 M NaOH, 30% v/v H_2_O_2_ for discovering the stability nature of the drug. The degraded samples were prepared by taking suitable aliquots of the drug solution, and then undertaking the respective stress testing procedures for each solution. After the fixed time period, the stressed test solutions were diluted with the mobile phase. For every stress condition, a solution of concentration 4 μg/mL of ADV was prepared. The specific stress conditions are described as follows. 

*A: Acidic degradation condition*

Acidic degradation was carried out by adding 1 mL of 0.1 M HCl, and after 40 min neutralizing the mixture by adding 0.1 M NaOH. 

**Table 1:** Results of forced degradation study of ADV (all experiments performed at room temperature

Stress Applied	Degradation (%)	RSD (%)
**0.001 M NaOH**	61.29	3.12
**0.1 M HCl**	60.26	9.32
**30% H**_2_ **O**_2_	7.93	6.63

**Table 2:** Intra and inter-day variations of the HPLC method for determination of ADV

Concentration (µg/mL)	Intra-day precision (%RSD)	Inter-day precision (%RSD)
**0.5**	10.3	7.25
**2**	6.3	8.65
**8**	2.4	3.68

**Table 3:** Results of accuracy/recovery of the proposed method

Concentration(µg/mL)	%Recovery	%R.S.D.
**3**	102.2	1.64
**6**	97.5	1.93
**12**	100.2	1.01

**Figure 2:**
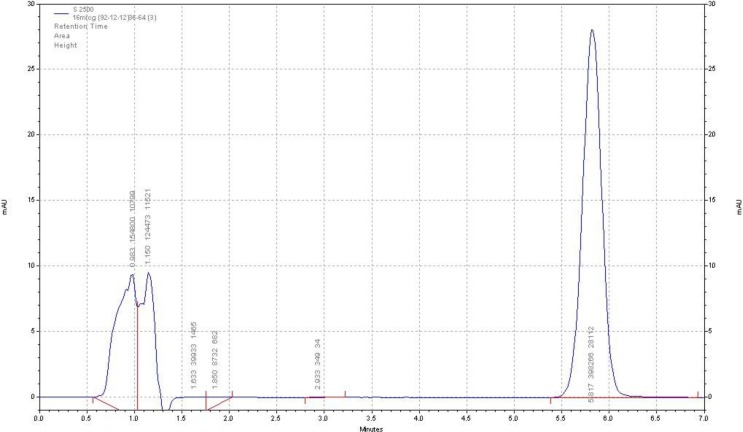
Chromatogram of adefovir dipivoxil drug substance (chromatographic condition described in the text

**Figure 3:**
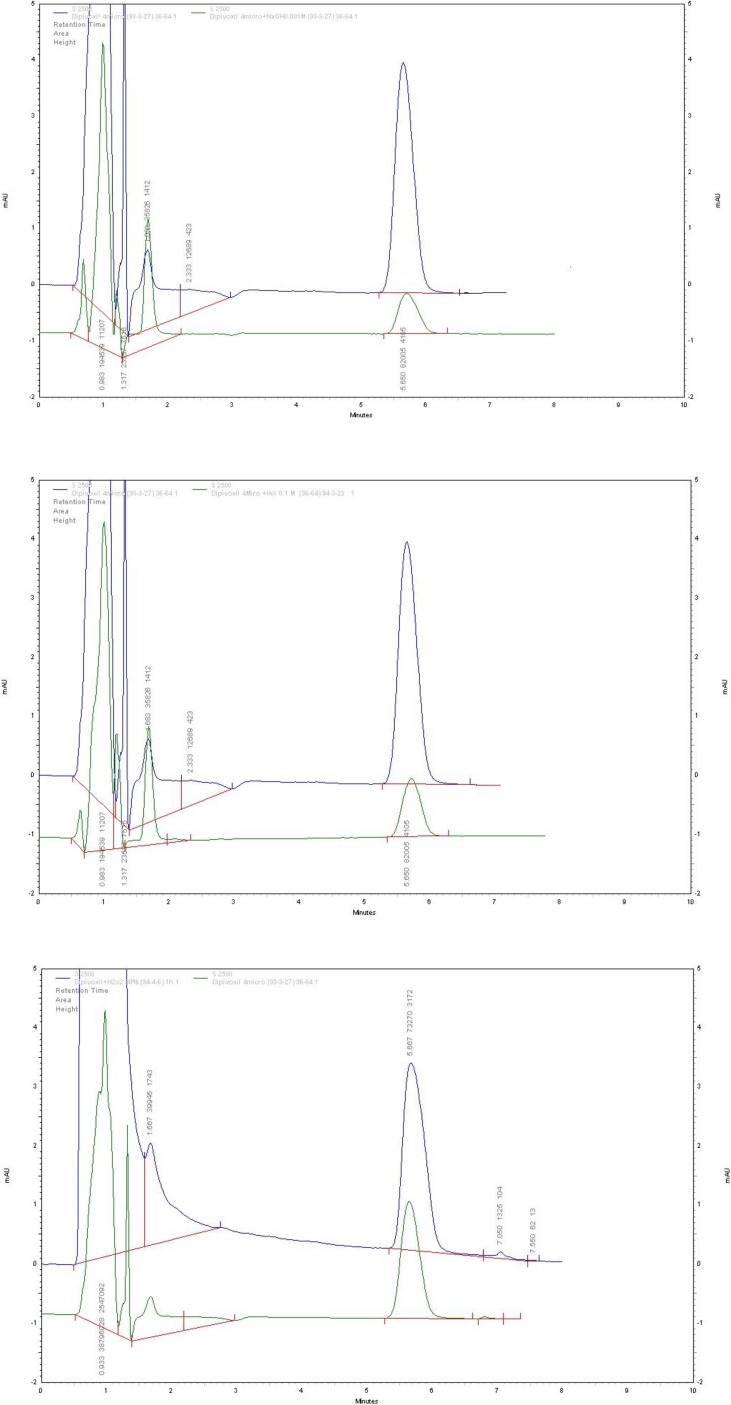
Chromatograms of ADV, before and after forced stress tests: (a) alkali-degraded drug; (b) acidic degraded drug; (c) oxidative degraded drug

**Figure 4:**
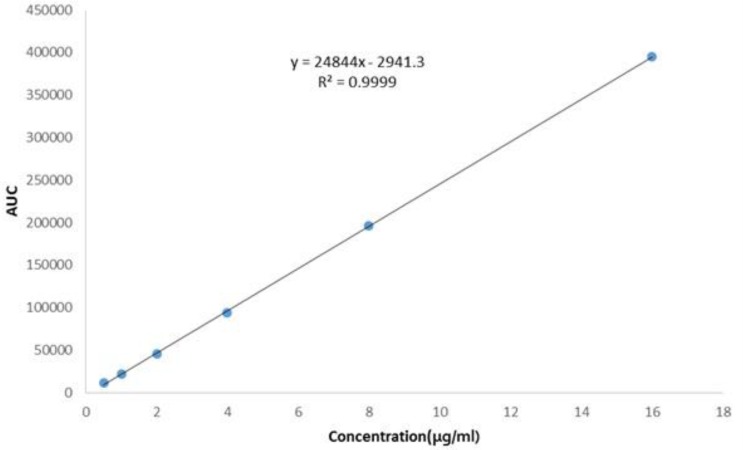
Linearity plot for ADV drug substance

*B: Alkali degradation condition*

Alkali total degradation was carried out by adding 1 mL of 0.1 M NaOH, leaving the mixture at room temperature for 45 min and neutralizing the mixture by adding 0.1 M HCl. Also, a milder basic condition, i.e. 0.001 M NaOH at room temperature for 5 min was applied to observe interferences from possible hydrolysis

*C: Oxidative degradation condition*

Oxidative degradation was performed by exposing the drug to 1 mL of 30% (v/v) H_2_O_2_ for 1 h.

*Method validation*

Validation of the proposed method was carried out according to the ICH guidelines (11). The assessment includes specificity, precision, linearity, accuracy, as well as determining LOD and LOQ.

*Specificity*

The specificity of the proposed method was assessed by checking the possible interferences from any products of the forced degradation of ADV. Also, any possible interference from the excipients of the commercial tablets was assessed. Ten mg of ADV was spiked with 10mgof excipient mix and the sample was analyzed for % recovery of ADV.

*Precision*

The precision was determined as both repeatability and intermediate precision by determining intra-day and inter-day variation, respectively, at 3 concentration levels of ADV (0.5, 2 and 8 μg/mL) in triplicate. 

*Linearity*

Linearity study was performed over a concentration range of 0.5–16 μg/mL of ADV at 0.5, 1, 2, 4, 8 and, 16 μg/mL. The prepared standard solutions were injected in series; each concentration level was analyzed three times. Mean of the peak area was calculated for each dilution and the obtained values were plotted against concentration.

*Accuracy*

The accuracy of the method was assessed by 3 replicate analysis of standard solutions of ADV at three concentrations of 3, 6, and 12 µg/mL, and then calculating each concentration from the calibration curve of the linearity assessment. 

*Detection and quantitation limits (sensitivit*y) 

Limit of quantification (LOQ) was determined during the evaluation of the linear range of calibration curve. LOQ was defined as the lowest concentration yielding a precision (%CV) and accuracy (% recovery) within their acceptable range with a peak area of three time limits of detection (LOD) (12).

## Results and Discussions

*Method development*

Assaying active pharmaceutical ingredients in bulk and dosage forms is essential for ensuring quality of the pharmaceutical products. It is routinely done by appropriate methods which must be simple, fast and reliable, as well as economic. The proposed HPLC method described in this paper for assaying ADV is fast, as the total analysis time is less than 7 min. It is also as simple as the RP-HPLC, which is now routinely in use in the pharmaceutical industry. The proposed chromatographic conditions applied an available C_18_ column with minimum use of the organic solvent, acetonitrile. The method also is fully validated in terms of precision, accuracy, and linearity over a reasonable concentration range of ADV. The method is free from interferences from any possible forced degradation products of the main compound. Thus, compared to other previously reported methods for assaying ADV in bulk and dosage forms, our proposed method is closer to the ideal conditions of an assaying method for routine industrial use. 

The HPLC procedure was optimized with a view for stability-indicating assay. During the trial runs, the drug was tested on an available C_18_ column with different mobile phase compositions like acetonitrile: buffer phosphate 10 mM (PH = 5.2), methanol: buffer phosphate 10 mM (pH = 5.2) and acetonitrile: buffer citrate 10 mM (pH = 5.2) at various compositions and flow rates. The mobile phase consisting of acetonitrile: buffer citrate 10 mM (pH = 5.2) and (36:64, v/v respectively) at a flow rate of 1.5 mL/min was selected as it presented a sharp and symmetric peak for ADV. The retention time for ADV was found to be 5.8 min. The run time was 7 min. The tailing factor for ADV was found to be 1.2. UV detection was carried out at 260 nm. The separation was carried out at room temperature. Figure 2 represents the chromatogram of the ADV standard drug.

*Validation of the method*

*Specificity*

ADV underwent complete degradation under the alkaline stress conditions by using 0.1 M NaOH solution for 45 min at room temperature. Thus, the stress conditions under basic solution were gradually set to milder conditions (i.e. 0.001 M NaOH solution and exposure time of 5 min) until moderate degradation of ADV was observed. In this way, instead of total degradation of the drug and its hydrolysates, only stepwise hydrolysis of ADV was observed. Oxidative stress test under exposure to a 3% solution H_2_O_2_ was failed, thus the concentration of H_2_O_2_ was gradually increased to 30% to observe oxidative degradation of ADV. ADV showed degradation in the order of alkali > acid > H_2_O_2_. Figure 3 represents typical chromatograms obtained for ADV after being subjected to alkali, acid, and H_2_O_2_ degradation conditions, respectively. There was no change in retention time of ADV. 

The obtained pure peaks suggested that there were no co-eluting or hidden peaks with the drug peak, which shows specificity and the stability-indicating nature of the method. The results for the forced degradation study are summarized in Table 1.

*Linearity*

The plot of peak area responses against concentration of ADV, obtained by regression method is shown in Figure 4. The plot is linear over the concentration range of 0.5-16 μg/mL yielding a regression equation *Y *= 24844*X *+ 2941.3 with a coefficient of correlation of 0.9999. 

*Precision*

The results obtained for repeatability studies and for intermediate precision are presented in Table 2. Method precision has a relative standard deviation (RSD) of 10.3% for repeatability and 8.65% for intermediate precision.

*Accuracy*

The results of accuracy assessment obtained for different ADV concentrations are shown in Table 3 which indicated the percent recoveries ranging from 97.5 to 102.2% with RSDs ranging from 1.01 to 1.93% which comply with the acceptance criteria proposed (% Recovery range: 80- 120%) (13). 

*Detection and quantitation limits (sensitivity)*

The results showed that the detection and quantitation limits for ADV using this method are 0.12 μg/mL and 0.35 μg/mL, respectively.

*Application of the method*

The developed HPLC method was applied for the determination of ADV in a commercial tablet dosage form containing 10 mg of the drug. The results of fifth analysis indicated that the amount of ADV was 9.81 ± 0.04 mg per tablet which was in accordance with the label 

claimed.

## Conclusions

The proposed HPLC method in this report is a rapid and easy stability-indicating method for assay of ADV. The method was proved to be specific, accurate, precise, and reproducible. The method was shown to use minimum amount of the organic modifier in the mobile phase, compared to similar previously reported methods. Thus, the method provides greater safety and environmental concerns. The method was successfully used, without interference from the degradation products and /or excipients, for routine analysis of ADV in bulk and tablet dosage forms. 
